# Application of ‘mobile hospital’ against 2019-nCoV in China

**DOI:** 10.1017/S0950268820000862

**Published:** 2020-04-24

**Authors:** Hai-ping Yu, Li-li Ma, Yun-ying Hung, Xue-bin Wang, You-qing Peng, chi Chen, Hui-ren Zhuang

**Affiliations:** 1 Department of Nursing, Shanghai East Hospital, Tongji University School of Medicine, Shanghai, China; 2 Nursing Director of Outpatient Department, Shanghai East Hospital, Tongji University School of Medicine, Shanghai, China; 3 Associate Director of Department of Nursing, Chung Hwa University of Medical Technology, Tainan, Taiwan; 4 Doctor Director of ICU，Shanghai East Hospital, Tongji University School of Medicine, Shanghai, China; 5 Department of Nursing, Shanghai East Hospital, Tongji University School of Medicine, Shanghai, China; 6 Director of Teaching and Training Department, Shanghai East Hospital, Tongji University School of Medicine, Shanghai, China; 7 Shanghai East Hospital, Tongji University School of Medicine, Shanghai, China

The World Health Organization (WHO) has declared the outbreak of the novel coronavirus (2019-nCoV) a public health emergency of international concern [1], which poses a great challenge to China's health system. As of 28 February 2020, the total number of confirmed COVID-19 patients had reached 78 961, with 2791 deaths [2]. To effectively control the spread of the epidemic and provide medical services, the government directed general hospitals to rapidly open fever clinics. More than 110 fever clinics are required to provide services to the public in Shanghai. However, due to the sudden outbreak of the epidemic and the arrival of the Spring Festival, most hospitals have been unable to cope with the consequences of the 2019-nCoV outbreak.

A mobile hospital is a kind of temporary medical institution that can be rapidly deployed [3], and is often used as a medical resource to rapidly implement treatment in case of an emergency. Our hospital has an International Emergency Medical Team, which is certified by the WHO and equipped with perfect mobile hospital facilities. In this anti-epidemic battle, our hospital adopted the mobile hospital to deal with patients in the early stage of the outbreak in Shanghai and as a supplement medical facility to assist the Wuhan KeTing Medical Shelter. Here, we describe the lessons learned during these deployments.

## As a temporary facility in the early stage of the outbreak

On 23 January, we completed the layout of the mobile hospital fever clinic in just 4 h, which not only effectively deals with fever patients, but also saves precious time for our hospital to complete the formal reconstruction of its fever clinic. From 23 to 27 January, 367 fever patients were registered in the mobile hospital, including one confirmed patient, without any medical staff being infected. Our experiences are described below.

### Establishing a multiple disciplinary management team

We established a management team, including experts in infection control management, medical and nursing administration, IT management, pharmacy, laboratory testing, material procurement and logistics management. The anti-epidemic management team was led by a hospital leader, and everyone performed their duties and cooperated sincerely (Table 1).

**Table 1.** Responsibilities of the various departments

Department	Responsibility
Infection control	1. Designing the layout of the mobile hospital 2. Providing epidemic prevention training for all personnel 3. Monitoring the protection and infection control
Logistics management	1. Rapid construction of a mobile hospital 2. Cleaning
Procurement	Rapid purchasing of required materials
Medical administration	1. Establishing a doctor team 2. Making diagnosis and treatment processes
Nursing administration	1. Human resource management of nurses 2. Making triaging and nursing processes 3. Daily material management
IT	1. Installation of the electronic information system 2. Monitoring and operation of the system
Laboratory	1. The layout of the laboratory 2. Sampling and testing
Pharmacy	1. The layout of pharmacy 2. Management of medicine
Patient service	Printing and posting all kinds of signs and health education materials
Security	Order maintenance

### Layout of the mobile hospital

**Fig. 1.**
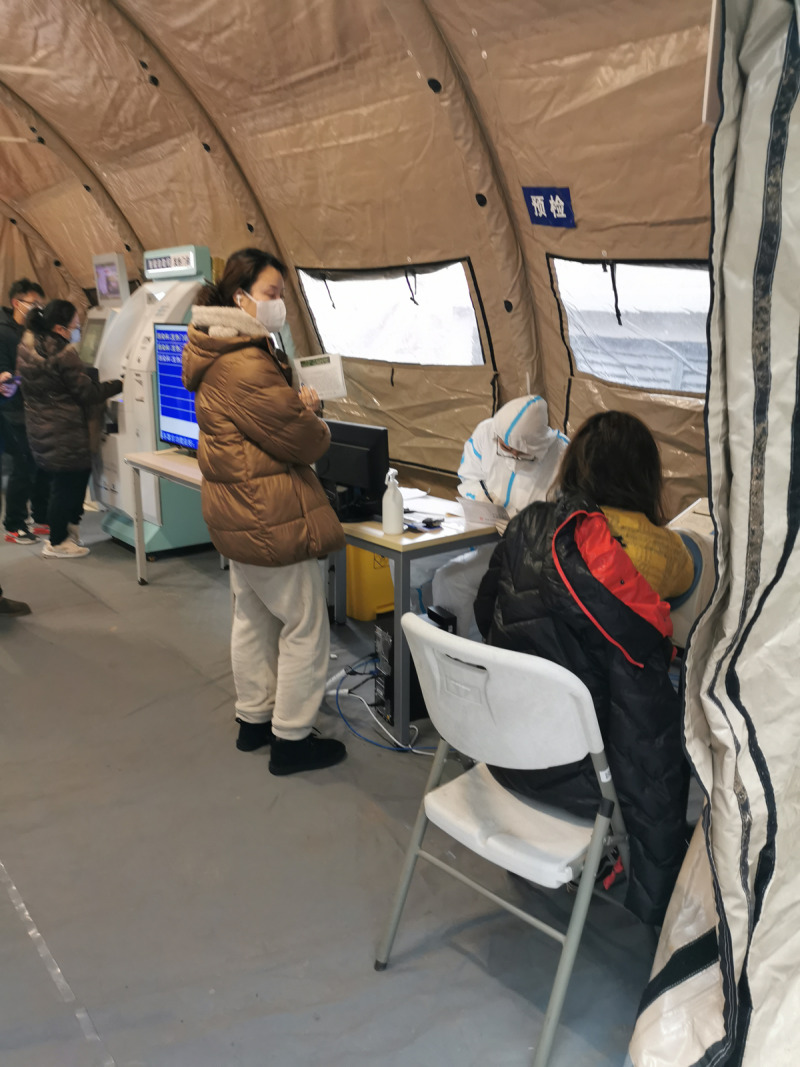
Triage.

**Fig. 2.**
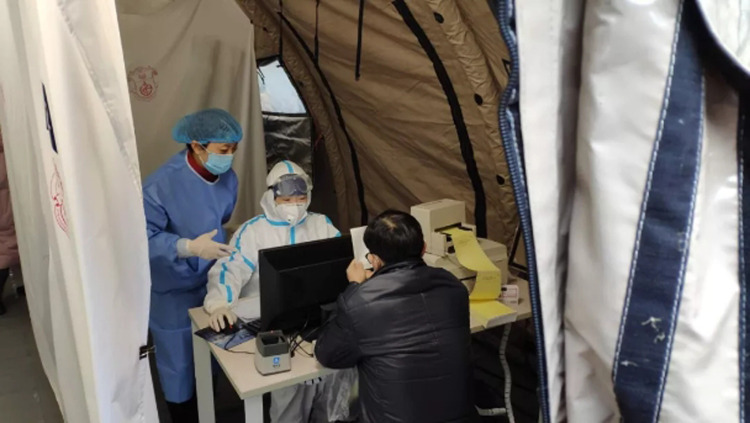
Consultation Room.

The layout of the mobile hospital was completed according to the requirements of infectious diseases, and consisted of 12 folding tents. Each tent covers an area of 48 m^2^ with separate negative pressure air purification systems. The following areas were set up: triage and waiting room, consultation room, laboratory, pharmacy, treatment room (equipped with six infusion chairs), two isolation wards (equipped with beds and mobile toilets) and rest and dressing rooms for medical staff (Figs 1 and 2).

### Operation management of the mobile hospital

All staff members were required to wear personal protective equipment. Each shift was staffed by two doctors, two nurses, one pharmacist, two laboratory technicians, one cleaner and one security guard. All staff in the mobile hospital were provided with level II protective equipment (protective clothing, goggles, N95 masks and gloves), and the laboratory technician was provided with level III protective equipment (as for level II, except that the goggles are replaced with a face mask).

Strictly enforced temperature measurements were implemented. To prevent cross-infection and ensure the accuracy of temperature measurements, the nurses used ear temperature guns to measure the temperature of patients, and used disposable earmuffs. If necessary, mercury thermometers were used for accurate thermometry.

The patients were required to provide complete personal information and were questioned about their epidemiological history, such as: Have you been to an epidemic area within the last 14 days? Have you contacted people from the epidemic area? Patients also signed a letter of commitment for the information they provided. Twenty waiting chairs were set up, 1 m apart. All patients and caregivers were required to wear masks, and the hospital provided masks free of charge to those without masks.

The patients were screened by using nasopharyngeal swab, blood sampling and lung computerised tomography (CT) scans. Anti-2019-nCoV experts in the hospital consulted about the suspected patients, and these patients were reported to the district health supervision institute. The mobile hospital was equipped to carry out samplings that were then sent for nucleic acid detection in the Center for Disease Control and Prevention. While awaiting the report, the patient received relevant treatment in the isolation room, and the hospital provided all daily necessities. Then, the confirmed patients were transferred to infectious disease hospitals using a special ambulance.

This outbreak occurred suddenly and during the Chinese Spring Festival, and few epidemic prevention materials were available. In the cleaning area of the mobile hospital, five cabinets were used for storing protective materials. The nurse manager was responsible and accountable for the management of these protective materials.

### Disinfection management

**Fig. 3.**
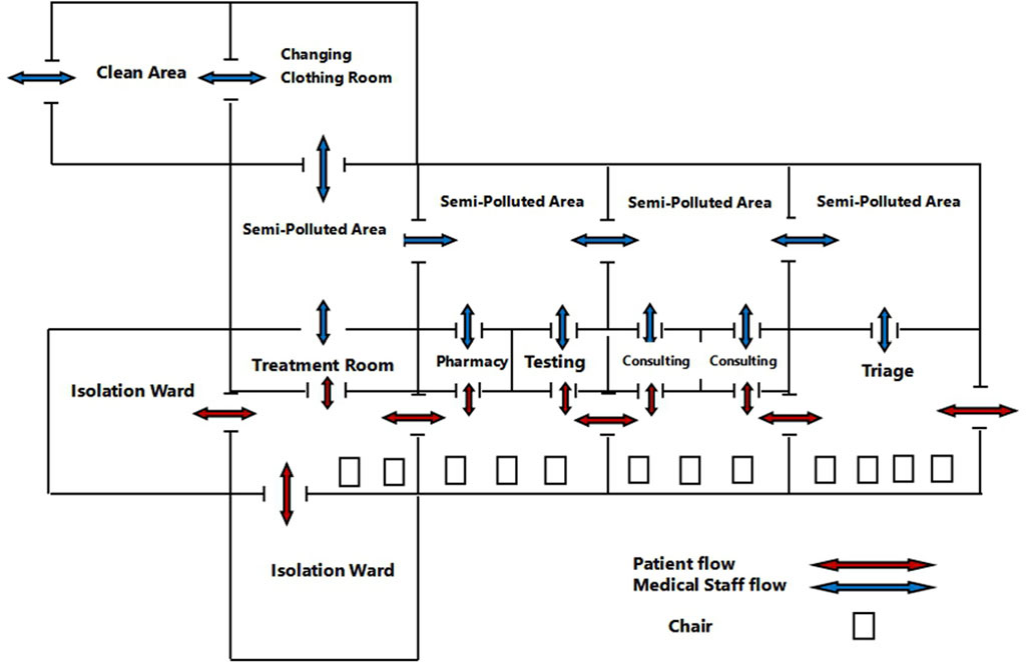
Schematic Diagram.

Given the flexibility of the mobile hospital, we were able to quickly and effectively set up three areas and two ‘channels’. Polluted areas, semi-polluted areas and a clean area were well divided, and two separate channels were built for patients and medical staff (Fig. 3).

A quick hand disinfectant facility was set up. Coronaviruses are sensitive to alcohol disinfectants, and alcohol hand disinfectants were placed on all tables and next to registration machines. Two ultraviolet disinfection lamps were fixed for air disinfection. Indoor articles were wiped with 2000 mg/l chlorine, and the ground was mopped with 1000 mg/l chlorine, every 2 h.

The vomitus excreta of the patients was treated as class I infectious disease excreta. The excreta were disposed of in the mobile toilet. Two layers of infectious storage bags were placed in the pedestal pan in advance. The excreta were pretreated with 50 000 mg/l effective chlorine. After pouring the effective chlorine, the mouth of the bags was sealed, and the bags disposed of using the delivery box after 4 h. The bed articles used by the patients were placed directly into the double-layer infectious storage bags, and chlorine (10 000 mg/l) poured into the bags. Double bags and double seals, which were marked, were handed over to the washing unit for treatment.

The final disinfection of the confirmed patient's room was done using spray disinfection and a hydrogen peroxide machine. Given the inadequate airtightness of the tent, the room was sealed with sealing strips before disinfection. The total amount and time needed to disinfect a room was calculated based on the room volume. For a tent with an area of 48 m^2^, 60 min of disinfection was required, followed by 60 min of ventilation.

## As an important supplementary medical resource of the Wuhan KeTing Medical Shelter

On 4 February, our international emergency medical team was ordered to assist the Wuhan KeTing Medical shelter hospital. The medical shelter was originally reconstructed from the Wuhan Culture and Art Center, with a total of 1461 beds. As of 17:00 on 21 February, it had received 1701 patients with mild disease. The clinical manifestations of the patients with mild disease were slight, or they had a fever, respiratory tract problems and other symptoms. The imaging findings showed ground glass, no dyspnoea and chest distress. Arbidol hydrochloride tablets combined with Chinese oral medicine was the main treatment method. If the patient's condition worsened beyond the capacity of the shelter hospital, staff of the headquarter were responsible for contacting the designated hospital and arranging 120 special vehicles for medical staff to transport patients to the designated hospital. (The designated hospital is the comprehensive hospital closest to the FangCang, for example, the designated hospital for KeTing FangCang is Jin Yin Tan hospital, 1 km away.) There was 1043 medical staff staying in this shelter, including 151 doctors, 841 nurses, 11 imagers, five pharmacists, two infection control experts and 33 laboratory technicians.

**Fig. 4.**
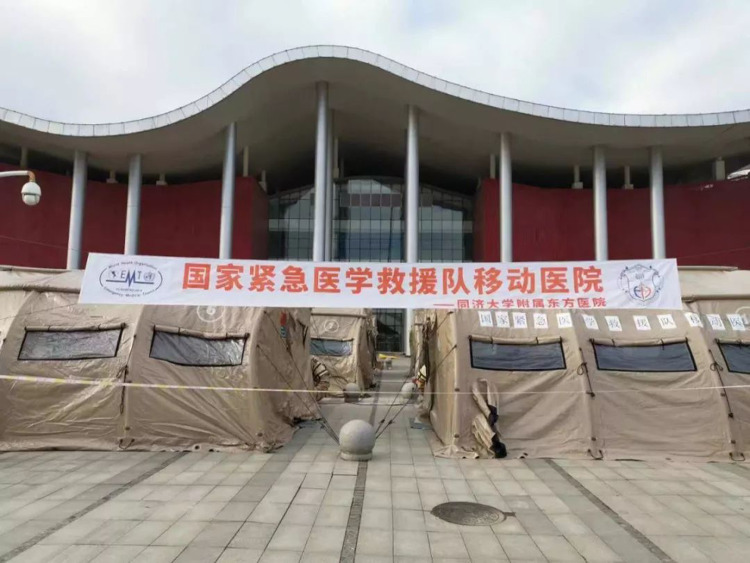
“Mobile Hospital” in Wuhan.

**Fig. 5.**
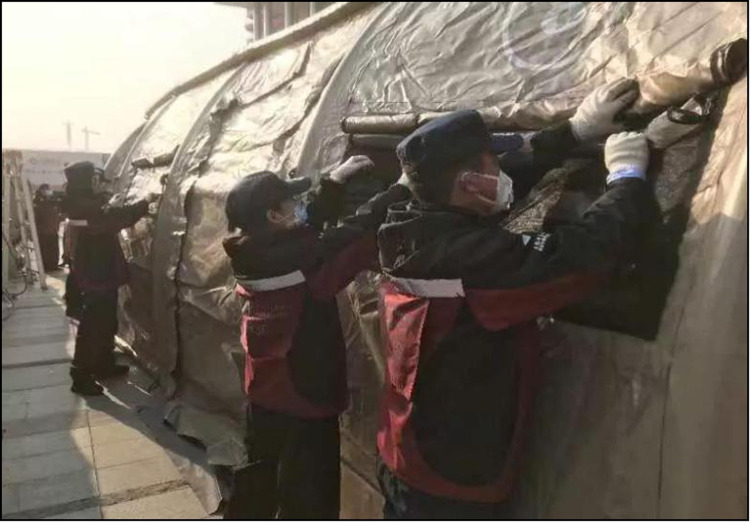
Setting up “Mobile Hospital” in Wuhan.

**Fig. 6.**
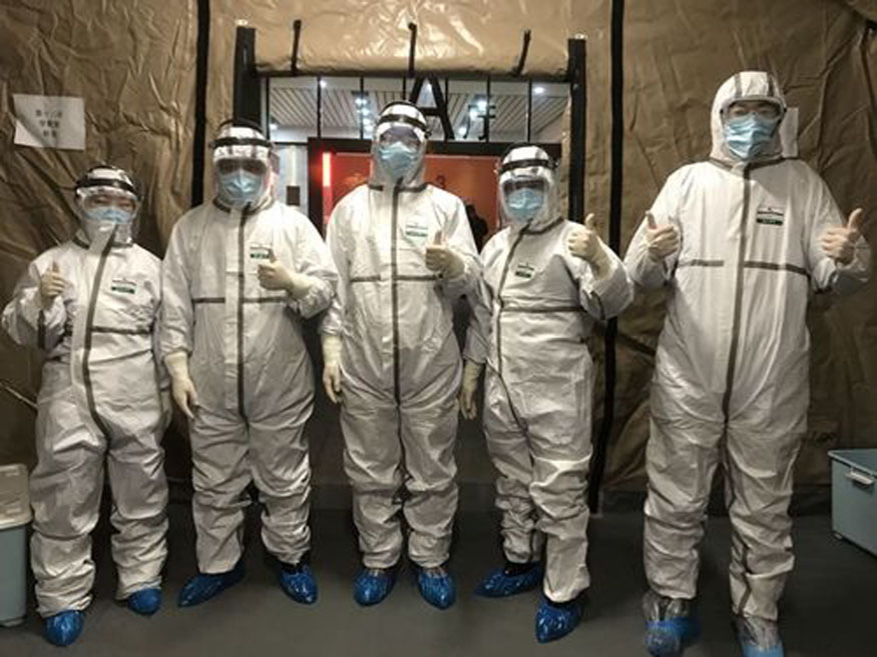
Tents used as the medical channels of “Shelter Hospital”.

Given that the shelter was reconstructed from an art centre, the layout of the medical facilities was imperfect. As soon as we arrived, our rescue team built a mobile hospital using 25 tents, including outpatient services, a pre-inspection space, observation rooms, monitoring rooms, a pharmacy, offices, meeting rooms, dressing rooms, restrooms, a canteen and bathrooms and toilets. Seven tents were used as the medical channels of the Shelter Hospital, which greatly contributed to the smooth operation of the hospital (Figs 4–6).

## Key lessons learned

**Fig. 7.**
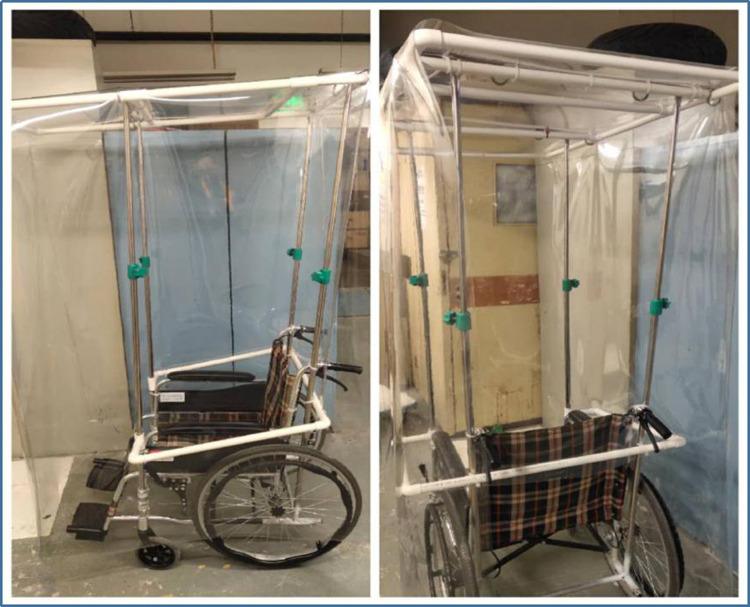
Transfer Wheelchairs.

**Fig. 8.**
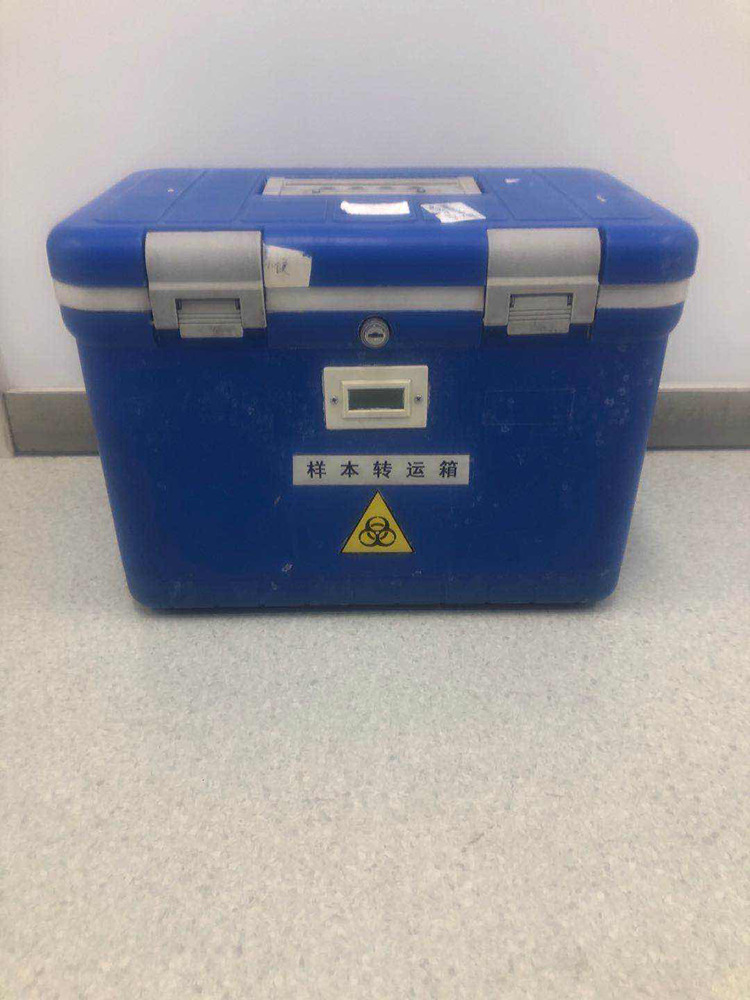
Specimen-transferring Box.

**Fig. 9.**
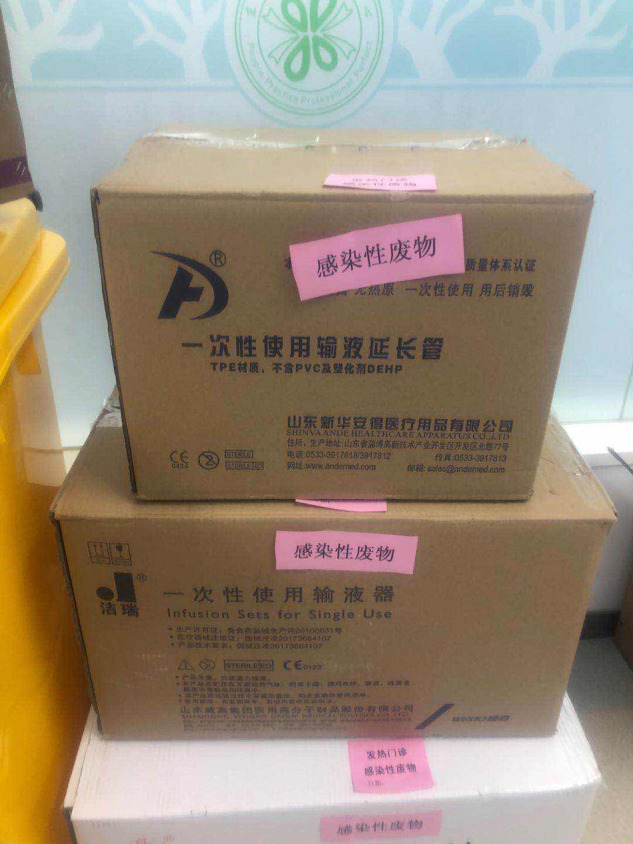
Sealed-discharge-transferring Boxes.

However, we also encountered many problems during implementation of the mobile hospital, including: how to supply water and power, how to transfer patients to receive CT examination (because it was impossible to perform CT scans in the mobile hospital) and how to transport patients' specimens and excretory wastes. To solve these problems, the following measures were taken: we chose to build the mobile hospital at a certain distance away from the main building, but close enough to connect a water source and power supply; we set up a special CT unit in the Emergency Department reserved only for those patients with fever, and also set up a special channel for patient transport to and from this CT unit with transfer wheelchairs (Fig. 7) and made use of special specimen-transferring boxes and sealed-discharge-transferring boxes (Figs 8 and 9).

**Fig. 10.**
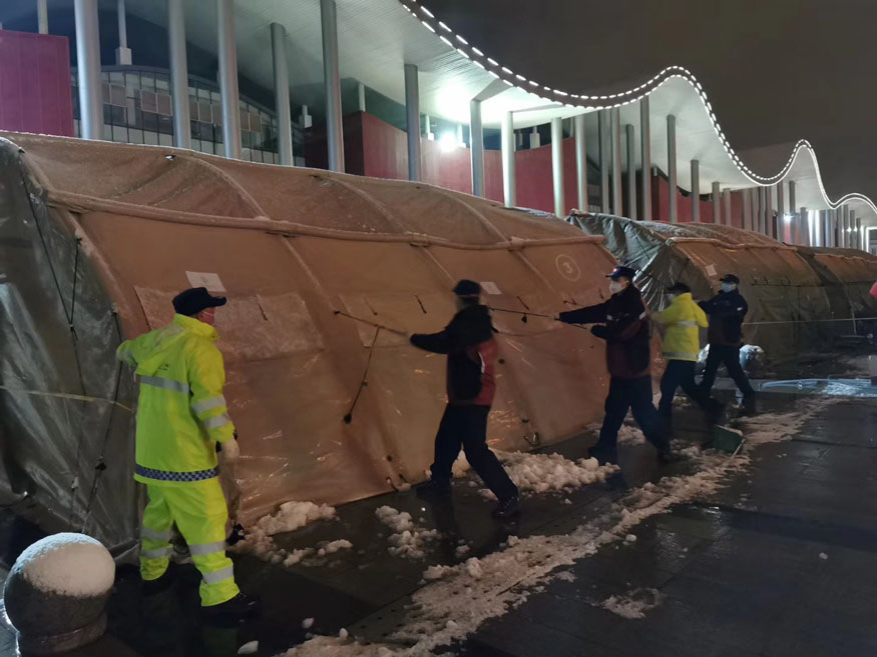
Cleaning up Snow in Wuhan.

Mobile hospitals have some shortcomings that need to be further improved. First, due to the thin material of the tent, the thermal insulation is poor, and given that air conditioning cannot be used (because this would assist in spreading the virus), the medical staff reported that they felt cold working in the tent. Also, there are certain security risks associated with the weather. The mobile hospital in Shanghai encountered 3 days of rain, and water needed to be cleared and anti-skid matting used. In Wuhan, there was heavy snow (Fig. 10), which needed to be cleared. How mobile hospitals can better cope with bad weather should be addressed in future work.

In conclusion, mobile hospitals, which are characterised by flexibility, have played a critical role in this anti-epidemic campaign. These mobile hospitals have been rapidly put into use, thereby alleviating the shortage of medical resources in the early stage of the anti-epidemic campaign.
